# Retinoids induce antagonism between FOXO3A and FOXM1 transcription factors in human oral squamous cell carcinoma (OSCC) cells

**DOI:** 10.1371/journal.pone.0215234

**Published:** 2019-04-12

**Authors:** Kwame Osei-Sarfo, Lorraine J. Gudas

**Affiliations:** 1 Department of Pharmacology, Weill Cornell Medical College, New York, NY, United States of America; 2 Weill Cornell Meyer Cancer Center, New York, NY, United States of America; University of Illinois at Chicago, UNITED STATES

## Abstract

To gain a greater understanding of oral squamous cell carcinoma (OSCC) we investigated the actions of all*-trans*-retinoic acid (RA; a retinoid), bexarotene (a pan-RXR agonist), and forkhead box (FOX) transcription factors in human OSCC-derived cell lines. RA and bexarotene have been shown to limit several oncogenic pathways in many cell types. FOXO proteins typically are associated with tumor suppressive activities, whereas *FOXM1* acts as an oncogene when overexpressed in several cancers. RA and/or bexarotene increased the transcript levels of *FOXO1*, *FOXO3A*, and *TRAIL* receptors; reduced the transcript levels of *FOXM1*, Aurora kinase B (*AURKB*), and vascular endothelial growth factor A (*VEGFA*); and decreased the proliferation of OSCC-derived cell lines. Also, RA and/or bexarotene influenced the recruitment of FOXO3A and FOXM1 to target genes. Additionally, *FOXM1* depletion reduced cell proliferation, decreased transcript levels of downstream targets of *FOXM1*, and increased transcript levels of *TRAIL* receptors. Overexpression of *FOXO3A* decreased proliferation and increased binding of histone deacetylases (HDACs) 1 and 2 at the *FOXM1*, *AURKB*, and *VEGFA* promoters. This research suggests novel influences of the drugs RA and bexarotene on the expression of *FOXM1* and *FOXO3A* in transcriptional regulatory pathways of human OSCC.

## Introduction

Oral squamous cell carcinomas (OSCCs) are a heterogeneous group of cancers that develop in the epithelial tissues of the tongue, hard and soft palate, retromolar trigone, gums, buccal mucosa, and lip [1]. At the end of 2017, the estimated new cases and deaths resulting from OSCC world-wide were 1,688,780 and 600,920, respectively [2]. The 5-year survival rate of OSCC has not significantly changed over the past few decades, despite advances in surgery, chemotherapy, and radiation [3, 4]. Initiation and development of OSCC have been linked to high consumption of tobacco and alcohol, viral infection, and poor oral hygiene [5, 6]. Thus, understanding the molecular signaling mechanisms that lead to OSCC is critical for the development of new therapies for OSCC.

The human forkhead box (FOX) gene family encodes transcription factors that are involved in multiple cellular processes, such as cell renewal and differentiation, cell proliferation, angiogenesis, immune regulation, DNA repair, and epigenetic modifications [7]. The members of this family have been categorized into 19 subgroups, based on homology outside and within the forkhead DNA-binding domain, and various family members are associated with the induction or suppression of several oncogenic signaling pathways [7, 8]. For instance, overexpression of *FOXM1* was reported in cancers of the breast, prostate, and lung [8]. We and others have shown increased *FOXM1* transcript and protein levels in the oral cavity during the development and progression of OSCC in both murine carcinogenesis models and human patient samples [9–13]. Additionally, FOXM1 is a prognostic factor for oral [14] and esophageal squamous cell carcinoma [15, 16]. The oncogenic effects of *FOXM1* generally are mediated through the phosphorylation of cyclin E-CDK2 and Raf-MEK-ERK signaling cascades that cause the nuclear translocation of FOXM1 [17, 18]. In the nucleus, FOXM1 can trigger the expression of several genes that are involved in tumor initiation processes such as angiogenesis, cell proliferation, cellular migration and invasion, and epithelial-mesenchymal transition [7]. FOXM1 also synergizes with the canonical *Wnt* signaling pathway (often activated during tumorigenesis) by directing the nuclear translocation of β-catenin to induce transcription of several oncogenes [19]. Additionally, increased *FOXM1* expression induces changes in the methylation status similar to the epigenome in OSCC [13]. Thus, *FOXM1* is a relevant target for further characterization because *FOXM1* regulates the expression of many genes and affects epigenetic controls that are involved in multiple oncogenic cellular processes.

In contrast to *FOXM1*, the members of the FOXO (*FOXO1*, *FOXO3A*, *FOXO4*, and *FOXO6*) gene family are considered to be negative regulators of the cellular events leading to tumorigenesis [20, 21]. Increased expression of *FOXO3A* reduces the oncogenic properties of cancers of the liver [22], lung [23], prostate [24], and oral cavity [25]. Molecular pathways implicated in cancer initiation that are inhibited by FOXO3A are similar to those increased by FOXM1 [26]. One mechanism by which FOXO3A exhibits its tumor suppressive properties is by transcriptionally antagonizing *FOXM1* [7, 27]. “Gain of function” p53 mutations induce *FOXM1* expression by inhibiting FOXO3A tumor suppressive signaling cascades [28]. Both FOXM1 and FOXO3A can alter transcription of target genes by binding to forkhead response elements (FHREs) on target promoters, which can result in opposing transcriptional outputs [29]. Additionally, differences among domains outside of the forkhead DNA binding domain in FOXM1 and FOXO3A result in recruitment of other proteins involved in modifying transcriptional events [7].

Retinoids (*e*.*g*. vitamin A, retinol) and derivatives (e.g. all*-trans*-retinoic acid, RA) are signaling molecules that regulate cell proliferation and differentiation [30]. RA alters cellular responses by binding to retinoic acid receptors α, β, or γ (RARs); the RARs and retinoid X receptors α, β, or γ (RXRs) form heterodimers that bind at retinoic acid response elements (RAREs) on target promoters [31, 32]. Various retinoids can also induce apoptosis in acute promyelocytic leukemia, T-cell acute lymphoblastic leukemia, myeloid leukemia, neoplastic epidermal keratinocytes, and breast cancer cells [33]. Bexarotene (Targretin, LGD1069), a pan RXR agonist, has been approved by the FDA for treatment of cutaneous T-cell lymphoma [34] and currently is in clinical trials for the treatment of metastatic breast cancer (Phase II) and non-small cell lung cancer (Phase II and III) [35].

Here, we analyzed the molecular actions of FOXM1 and FOXO3A in regulating gene expression, modifying oncogenic signaling pathways, and recruiting histone deacetylases (HDACs) in human OSCC cell lines. We also characterized the biological and molecular effects of RA and bexarotene on the proliferation of OSCC lines, the expression of FOX transcription factors, and the recruitment of FOXM1 and FOXO3A to FHREs on target promoters.

## Results

### Retinoic acid and bexarotene alter the mRNA levels of forkhead box genes and their downstream targets

**Fig 1:**
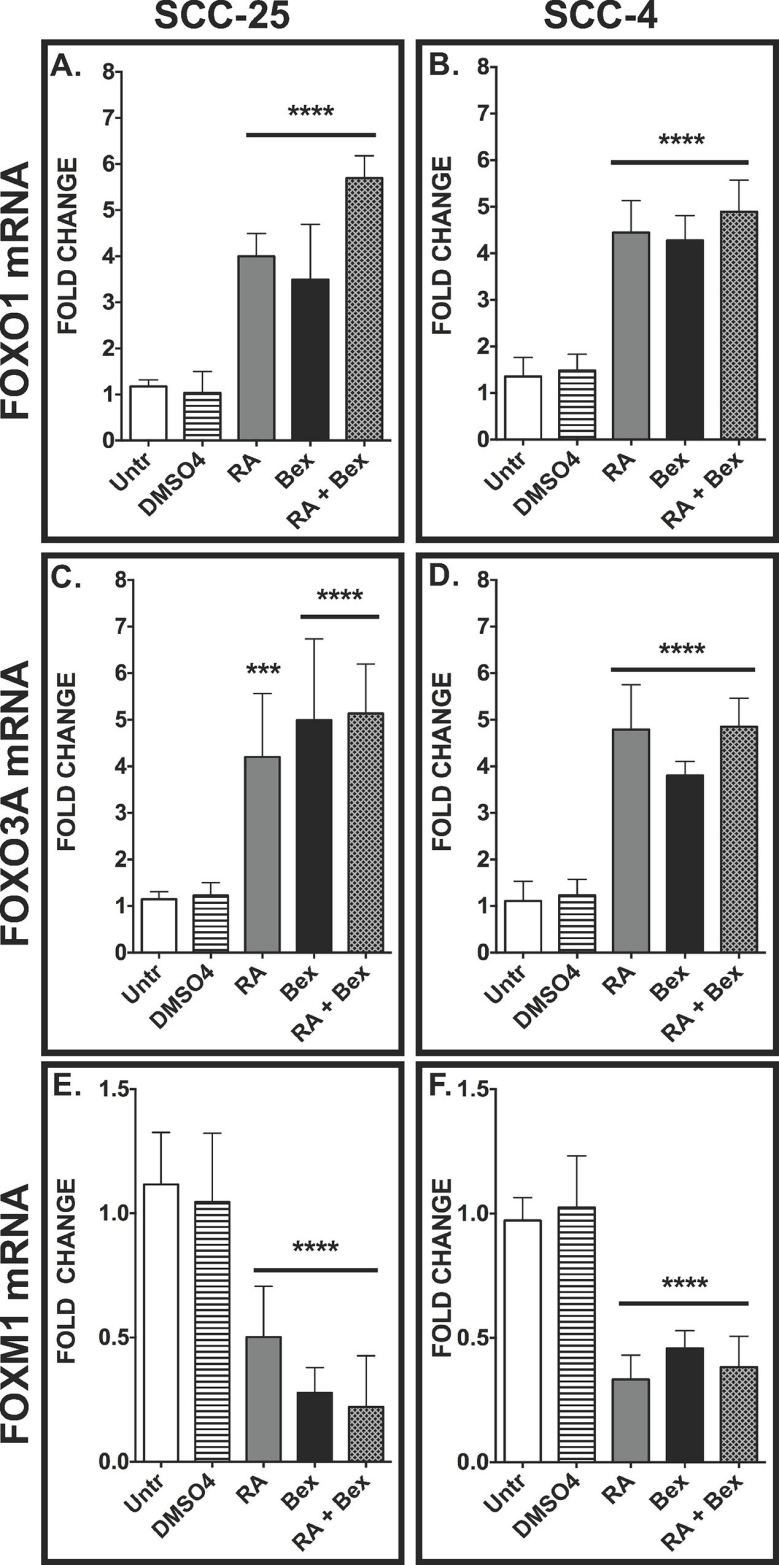
Retinoic acid (RA) and bexarotene (Bex) alter mRNA levels of FOXO1, FOXO3A, and FOXM1 in human OSCC cell lines. SCC-25 (A, C, and E) and SCC-4 (B, D, and F) cells were treated with nothing (Untr), 0.1% DMSO_4_ vehicle, either RA or Bex (final concentration of 1 μM and 10 μM, respectively), or the combination of RA plus Bex (RA+Bex). Quantitative Real-Time PCR (QRT-PCR) analysis was used to determine the transcript levels of *FOXO1* (A, B), *FOXO3A* (C, D), and *FOXM1* (E, F). The bars represent the means of five independent experiments ± SEM where ***, *p*<0.001 and ****, *p*<0.0001.

To determine if retinoic acid (RA), bexarotene (Bex), or the co-administration of RA plus bexarotene (RA+Bex) changed the transcript levels of various FOX transcription factors, we quantified the changes in *FOXO1*, *FOXO3A*, and *FOXM1* in the SCC-25 and SCC-4 human cell lines by QRT-PCR (Fig 1). We measured 3.5 to 5.8 fold increases (*p*<0.0001) in the *FOXO1* transcript levels in RA, Bex, and RA+Bex treated SCC-25 (Fig 1A) and SCC-4 (Fig 1B) cells compared to untreated (Untr) cells. We observed similar increases in the *FOXO3A* transcript levels in SCC-25 (Fig 1C) and SCC-4 (Fig 1D) treated with RA, Bex, and RA+Bex. In contrast, we detected >50% decreases (*p*<0.001) in *FOXM1* mRNA in both SCC-25 (Fig 1E) and SCC-4 lines (Fig 1F) in all three (RA, Bex, and RA+Bex) groups. We did not observe any synergistic effects with the co-administration of both drugs (Fig 1).

**Fig 2:**
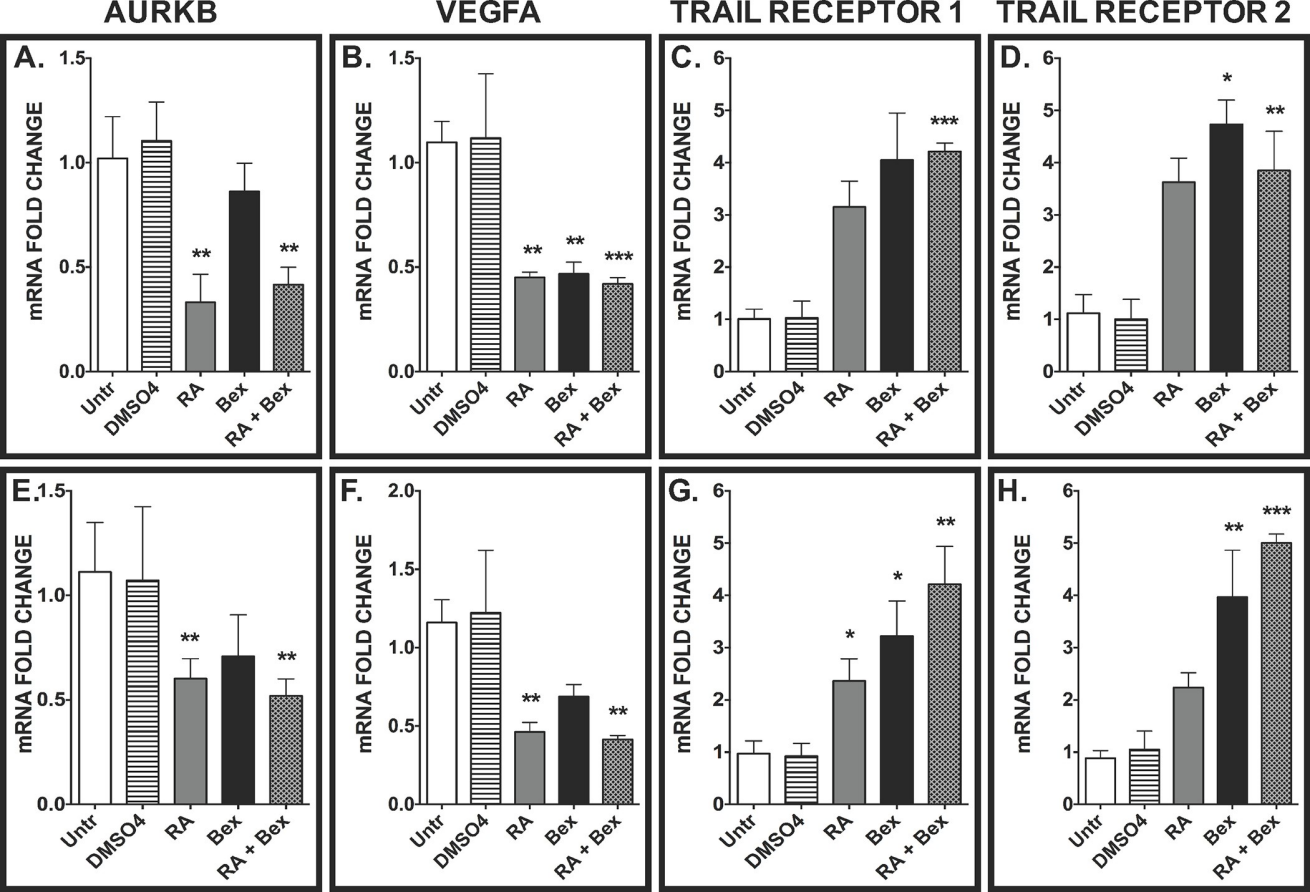
Treatment of OSCC lines with RA and bexarotene changes the transcript levels of downstream targets of FOX transcription factors. We used QRT-PCR to measure the transcript levels of Aurora kinase B (A, E), vascular endothelial growth factor A (VEGFA) (B, F), TNF-related apoptosis-inducing ligand (TRAIL) receptor 1 (C, G), and TRAIL receptor 2 (D, H) in the SCC-25 (A-D) and SCC-4 (E-H) lines treated with drugs as in Fig 1. Bars represent the means of four independent experiments ± SEM. Post-hoc test analyses show that **, ***, and **** represent *p*<0.01, *p*<0.001, and *p*<0.0001, respectively.

We next measured the transcript levels of well-characterized downstream targets of FOXO and FOXM1 transcription factors. We focused on the *FOXO3* subfamily because FOXO3A was shown to regulate the transcription of FOXO1 in human OSCC, suggesting that FOXO1 is downstream of FOXO3 signaling [36]. Downstream targets of FOXM1 and FOXO3A include Aurora kinase B (*AURKB*), vascular endothelial growth factor A (*VEGFA*), and TNF-related apoptosis-inducing ligand (*TRAIL*) receptors 1 and 2 [7, 37] in SCC-25 (Fig 2A–2D) and SCC-4 (Fig 2E–2H). We measured 2–4 fold decreases in the *AURKB* transcript levels (*p*<0.01) in SCC-25 and SCC-4 cells treated with RA and RA+Bex, but not with bexarotene alone (Fig 2A and 2E), suggesting that the changes in the *AURKB* transcript levels are mediated by RA. We found decreases (≥50%) in *VEGFA* transcripts in RA (*p*<0.01), Bex (*p*<0.01), and RA+Bex (*p*<0.001) treated SCC-25 cells compared to the untreated group (Fig 2B). We also detected decreases in *VEGFA* transcript levels in the RA (*p*<0.01) and RA+Bex (*p*<0.01) treatment groups in SCC-4 (Fig 2F). The transcript levels of *TRAIL* receptors 1 and 2 were substantially increased (>3-fold, >2-fold, respectively) in SCC-25 (Fig 2C and 2D) and SCC-4 (Fig 2G and 2H) cells. We did not observe additive effects when comparing RA or Bex treatment groups to the RA+Bex group (Fig 2).

### Bexarotene and RA reduce cell proliferation in SCC-25 and SCC-4 cells

**Fig 3:**
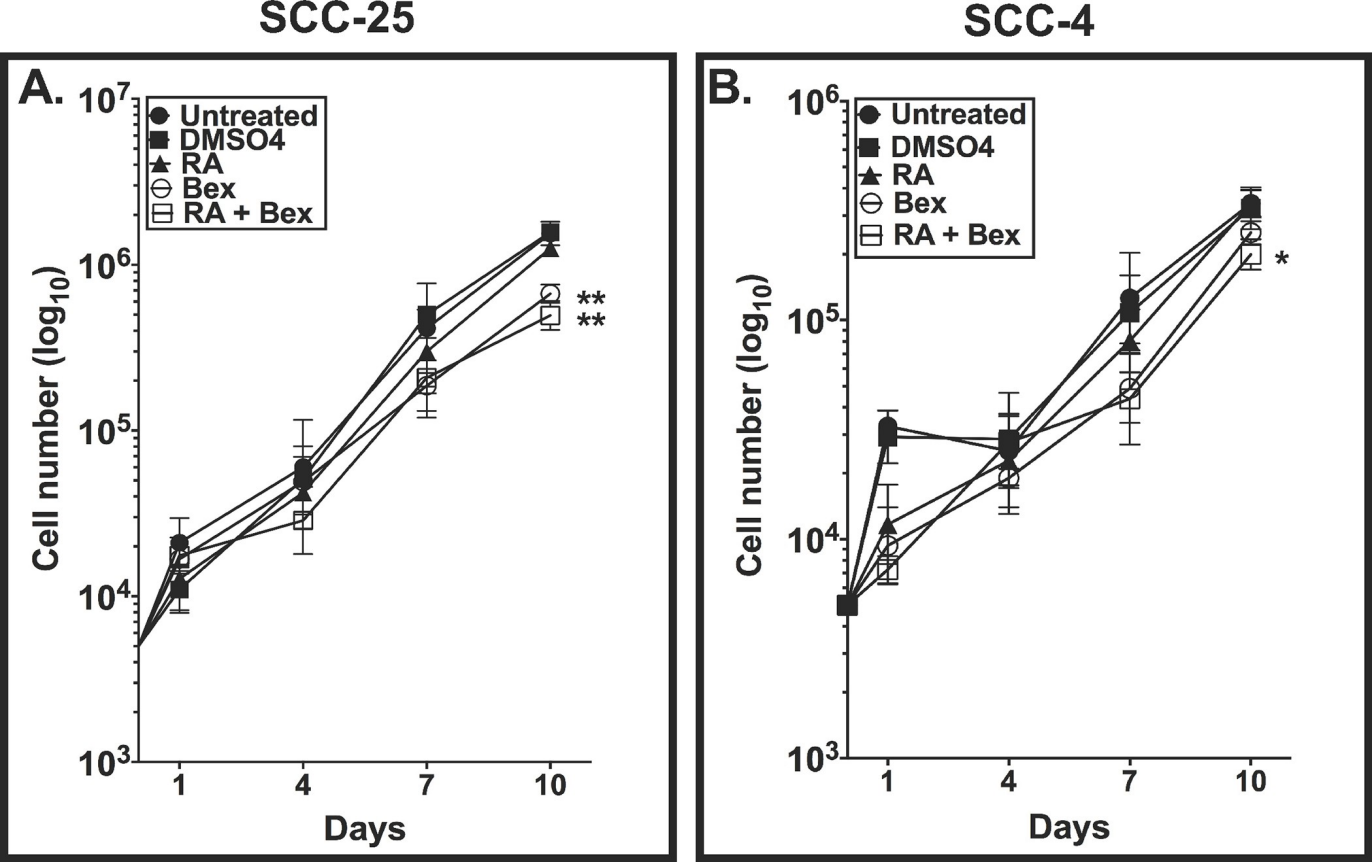
Bexarotene with RA reduces cell proliferation in OSCC-derived cell lines. Cell proliferation assays were conducted on SCC-25 (A) and SCC-4 (B) cell lines by seeding 5x10^3^ cells at Day 0. SCC-25 and SCC-4 cell lines were treated with 0.1% DMSO_4_ (vehicle control), RA, Bex, RA+Bex and counted at days 1, 3, 4, 7 and 10 (A, B). Statistical analysis was performed, using two-way ANOVA, followed by Bonferroni’s post-hoc test, on three independent experiments. Post-hoc test analysis show that * and ** represent *p*<0.05, *p*<0.01, respectively.

Bex and RA+Bex reduced the proliferation of both the SCC-25 and SCC-4 lines (Fig 3). At day 10 (the final time point for the cell proliferation assay), there were ~5.0x10^5^ SCC-25 cells in the Bex (*p*<0.01) and in the RA+Bex (*p*<0.01) groups, compared to ~1.5x10^6^ SCC-25 cells in the untreated group. In the SCC-4 cells, Bex (~2.5x10^5^ cells) and RA+Bex (~2.0x10^5^ cells) caused a smaller but statistically significant reduction in proliferation (*p*<0.05) versus untreated cells (~3.0x10^5^ cells) (Fig 3). In summary, RA plus bexarotene, compared to sole administration of RA or Bex, demonstrated a greater reduction in the proliferation of both OSCC-derived cell lines.

### Overexpression of exogenous FOXO3A and reduced expression of FOXM1 have similar effects on cell proliferation and target gene expression

**Fig 4:**
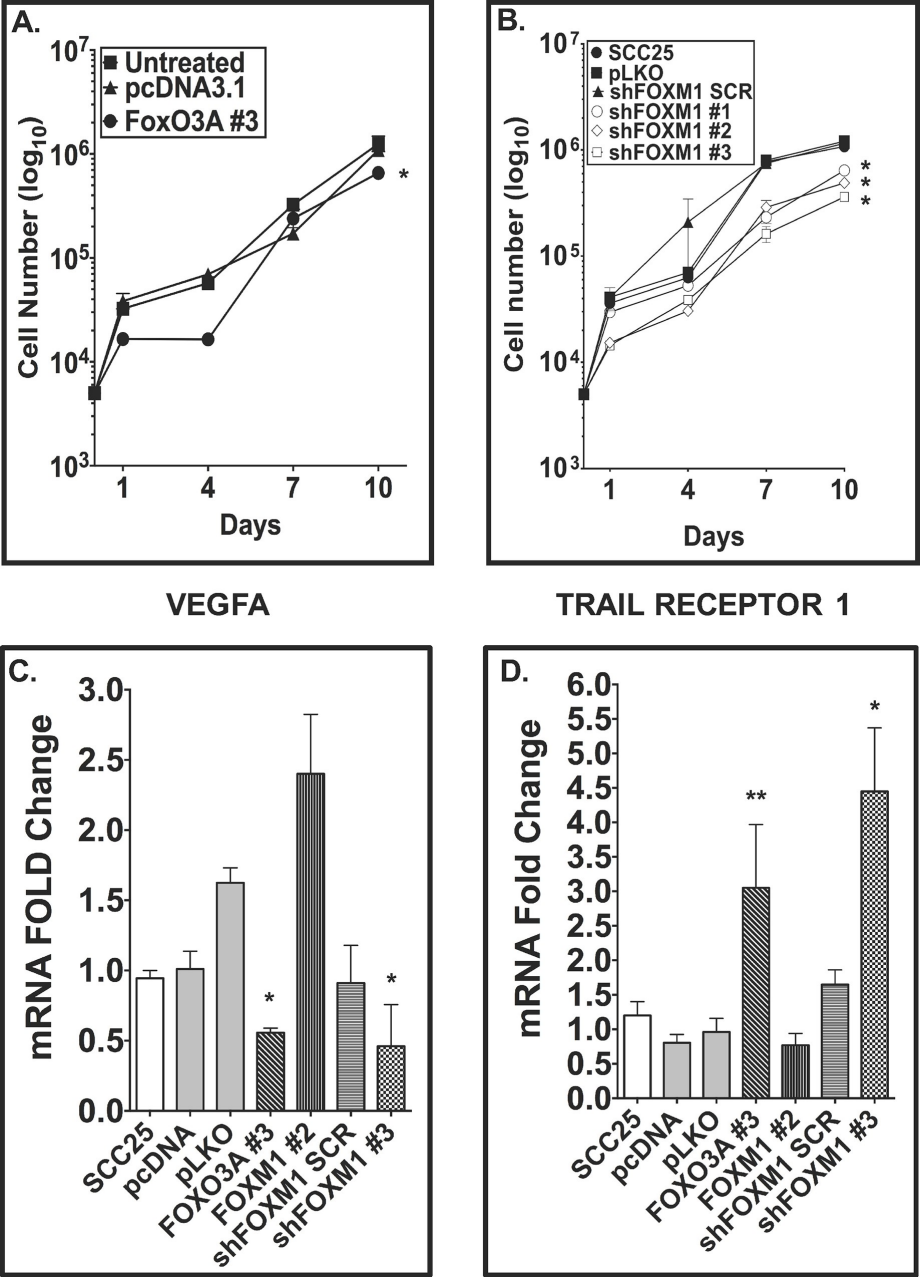
The overexpression of FOXO3A and the silencing of FOXM1 reduce cell proliferation and change expression of downstream FOX targets. Cell proliferation assays for SCC-25 cells (parental line) expressing exogenous FOXO3A (A) or shRNA constructs targeting FOXM1 (B) were conducted as described in Fig 3 with the quantitation of cell proliferation at days 1, 4, 7, and 10. Two-way ANOVA and Bonferroni’s post-hoc test were conducted to determine statistical significance on three independent experiments. The changes in the transcripts of VEGFA (C), and TRAIL receptor 1 (D) in SCC-25 cells expressing exogenous FOXO3A, exogenous FOXM1, and shRNA constructs targeting FOXM1 were determined by QRT-PCR analysis. The QRT-PCR data show the results of three independent experiments ± SEM (C, D). Post-hoc analyses show *, *p*<0.05 and **, *p*<0.01.

Several studies have demonstrated a connection linking the expression of microRNAs (miRNAs), the inhibition of FOXO3A, and the increased proliferation of cancer-derived cells: hepatocellular carcinoma (HCC)/miR-155 [38], HCC/miR-182-5p [39], and glioma/miR-155 [40]. Since reduced levels of FOXO3A increased cell proliferation in these cancer-derived cell lines, we wanted to determine if FOXO3A overexpression would reduce cell proliferation in an OSCC-derived cell line. After generating several SCC-25 cell clones with stable expression of exogenous FOXO3A or just with the empty vector pcDNA3.1 (S1A Fig), we found a minor reduction (*p*<0.01) in proliferation mediated by the SCC-25 FOXO3A #3 clone compared to the parental SCC-25 line (Fig 4A).

Since FOXM1 overexpression induces neoplastic transformation of breast epithelia [41], prostate epithelia [42], non-small cell lung cancer-derived cells [43], and oral cavity and esophageal epithelia [11, 14], we silenced FOXM1 expression by introducing FOXM1-targeting short-hairpin RNAs (shRNA) into SCC-25 cells (S1B Fig). We conducted cell proliferation assays of the SCC-25 parental line, SCC-25 cells that stably express the empty vector pLKO, SCC-25 cells that express a scrambled FOXM1 shRNA construct (shFOXM1 SCR), and SCC-25 cells that express a shRNA construct targeting FOXM1 (shFOXM1 #3) (Fig 4B). We found that the reduction in FOXM1 levels by shRNA silencing technology resulted in a reduction in cell proliferation (*p*<0.05) in an OSCC-derived cell line (Fig 4B).

To determine if FOXO3A overexpression and/or if the reduction in FOXM1 expression changed target transcript levels, we measured the transcript levels of VEGFA (Fig 4C) and TRAIL receptor 1 (Fig 4D). We detected a reduction in VEGFA mRNA in both SCC-25 cells that overexpress FOXO3A (clone SCC-25 FOXO3A #3), and in SCC-25 cells in which FOXM1 is silenced (clone SCC-25 shFOXM1 #3), compared to the parental SCC-25 cells, the cells stably transfected with empty vectors (pcDNA and pLKO), and cells transfected with the scrambled shRNA (SCC-25 shFOXM1 SCR). We detected increased transcript levels of the TRAIL receptor 1 (~3.0 fold, FOXO3A #3; ~4.8 fold, shFOXM1 #3) (Fig 4D). The overexpression of FOXO3A and the reduction in expression of FOXM1 both reduced Aurora kinase B transcript levels and increased TRAIL receptor 2 transcript levels (S1C and S1D Fig). These results confirm that ectopic over-expression of FOXO3A can reduce the proliferation of SCC-25 cells (Fig 4A), lower the expression of FOXM1 downstream effectors involved in cellular transformation (Fig 4C and S1C Fig), and induce expression of pro-apoptotic genes (Fig 4D and S1D Fig). Likewise, the silencing of FOXM1 (a putative oncogene in OSCC) by shRNA technology mimics effects on transcript levels seen by overexpressing FOXO3A in SCC-25 cells (Fig 4B–4D). Conversely, overexpression of FOXM1 (SCC-25 FOXM1 #2) in SCC-25 cells increased target gene transcripts involved in tumorigenesis (VEGFA and AURKB; Fig 4C and S1C Fig) and decreased those related to tumor suppression (TRAIL Receptor 1 and 2; Fig 4D and S1D Fig).

### RA and bexarotene induce changes in the binding of FOXM1 and FOXO3A at target gene promoters

**Fig 5:**
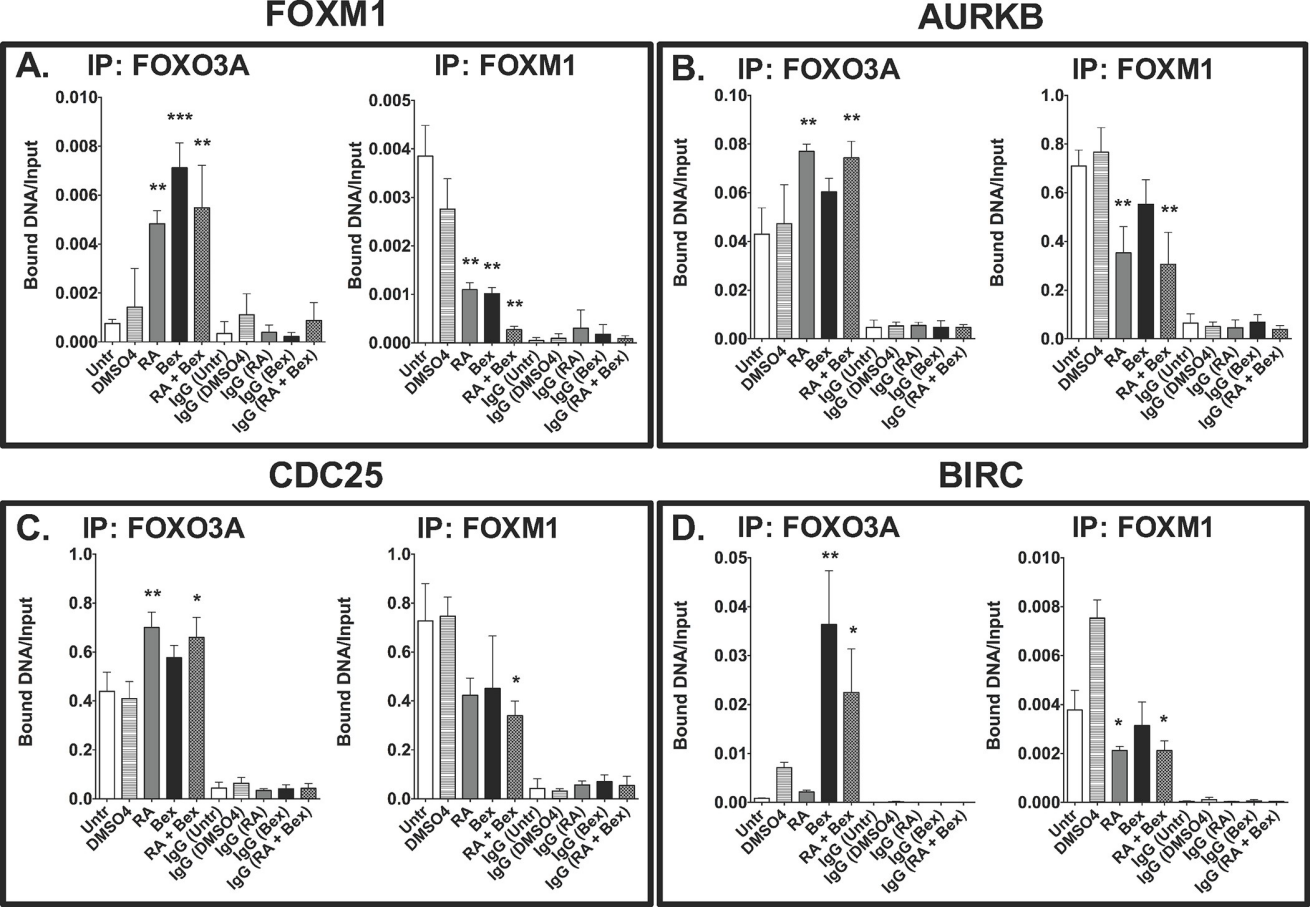
Administration of RA and/or bexarotene modifies promoter association of FOXO3A and FOXM1 transcription factors in SCC-25 cells. ChIP assays were performed to determine changes in the recruitment of FOXO3A and FOXM1 to target promoters in SCC-25 cells treated with vehicle (DMSO_4_), RA, Bex, and RA+Bex for 72 hours. Antibodies directed against FOXO3A, FOXM1, and IgG (negative control) were used to immunoprecipate soluble protein/chromatin complexes. Then, QRT-PCR was performed to amplify promoters of FOXM1 (A), Aurora kinase B (B), CDC25 (C), and BIRC (D). The bars signify the ratio of the PCR signal of the FOXO3A antibody, FOXM1 antibody, or IgG-bound DNA to that of the DNA input. These data represent the means of five independent experiments (biological repeats) ± SEM (*, *p*<0.05, **, *p*<0.01, and ***, *p*<0.001).

We carried out chromatin immunoprecipitation (ChIP) assays in the parental SCC-25 line to determine if FOXO3A competes with FOXM1 at specific promoters (Fig 5), similar to previous studies demonstrating FOXO1/FOXM1 binding [7]. We assayed the presence of FOXO3A and FOXM1 on the promoters of FOXM1, Aurora kinase B, CDC25, and baculoviral IAP Repeat containing 5 (BIRC5; also known as apoptosis inhibitor 4) +/- Bex and RA (Fig 5). On the FOXM1 promoter we detected increases in FOXO3A (measured by the ratio of bound DNA to input DNA) of 4–6 fold in SCC-25 cells treated with RA (*p*<0.01), Bex (*p*<0.001), and RA+Bex (*p*<0.01) (Fig 5A, left panel). We also observed approximately a 1.5-fold increase in the binding of FOXO3A at the Aurora kinase B promoter in RA (*p*<0.01) and RA+Bex (*p*<0.01) treated cells relative to untreated or DMSO_4_ (vehicle) treated cells (Fig 5B, left panel). Treatment with RA (*p*<0.01) and RA+Bex (*p*<0.05) increased the levels of bound FOXO3A on the mitotic regulator CDC25 promoter by ~1.5 fold (Fig 5C, left panel). We detected more FOXO3A at the BIRC5 promoter in the Bex (~0.035 bound DNA/Input; *p*<0.01) and the RA+Bex (~0.2 bound DNA/Input; *p*<0.05) treatment groups compared to the untreated and DMSO vehicle treated cells (~0.1 bound DNA/Input) (Fig 5D, left panel). The IgG levels (negative controls) in the samples were much lower than any of the FOXO3A antibody precipitated samples (Fig 5).

Conversely, the RA, Bex, and RA+Bex treatment groups exhibited lower FOXM1 binding at the FOXM1 promoter compared to the untreated and DMSO_4_ vehicle treated groups (~0.004); the ratios of bound DNA to the input DNA for these three treatment groups were ~0.001 (RA, *p*<0.01; Bex, *p*<0.01), and ~0.0001 (RA+Bex; *p*<0.001) (Fig 5A, right panel). We detected reductions in the binding of FOXM1 at the AURKB promoter in SCC-25 cells treated with either RA (*p*<0.01) or with RA+Bex (*p*<0.01), but not with Bex (Fig 5B, right panel). We detected a two-fold decrease in FOXM1 protein (~0.35 bound DNA/Input; *p*<0.05) at the CDC25 promoter in RA and RA+Bex treated as compared to the untreated or DMSO_4_ treated SCC-25 cells (~0.7 bound DNA/Input) (Fig 5C, right panel). The bound DNA/Input of FOXM1 at the BIRC5 promoter was ~0.002 for both the RA and the RA+Bex treatment groups (p<0.05; Fig 5D, right panel) compared to ~0.004 for the untreated cells.

Thus, these ChIP assays demonstrate that treatment with RA+Bex modified the binding of both FOXO3A and FOXM1 at the promoters of the FOXM1, Aurora kinase B, CDC25, and BIRC5 genes (Fig 5). In addition, the increased binding of FOXO3A and the decreased binding of FOXM1 at these target gene promoters after RA+Bex addition may result from the changes in FOXO3A and FOXM1 transcript levels induced by RA and/or bexarotene (Fig 1).

### The overexpression of exogenous FOXO3A induces recruitment of histone deacetylase (HDACs)

**Fig 6:**
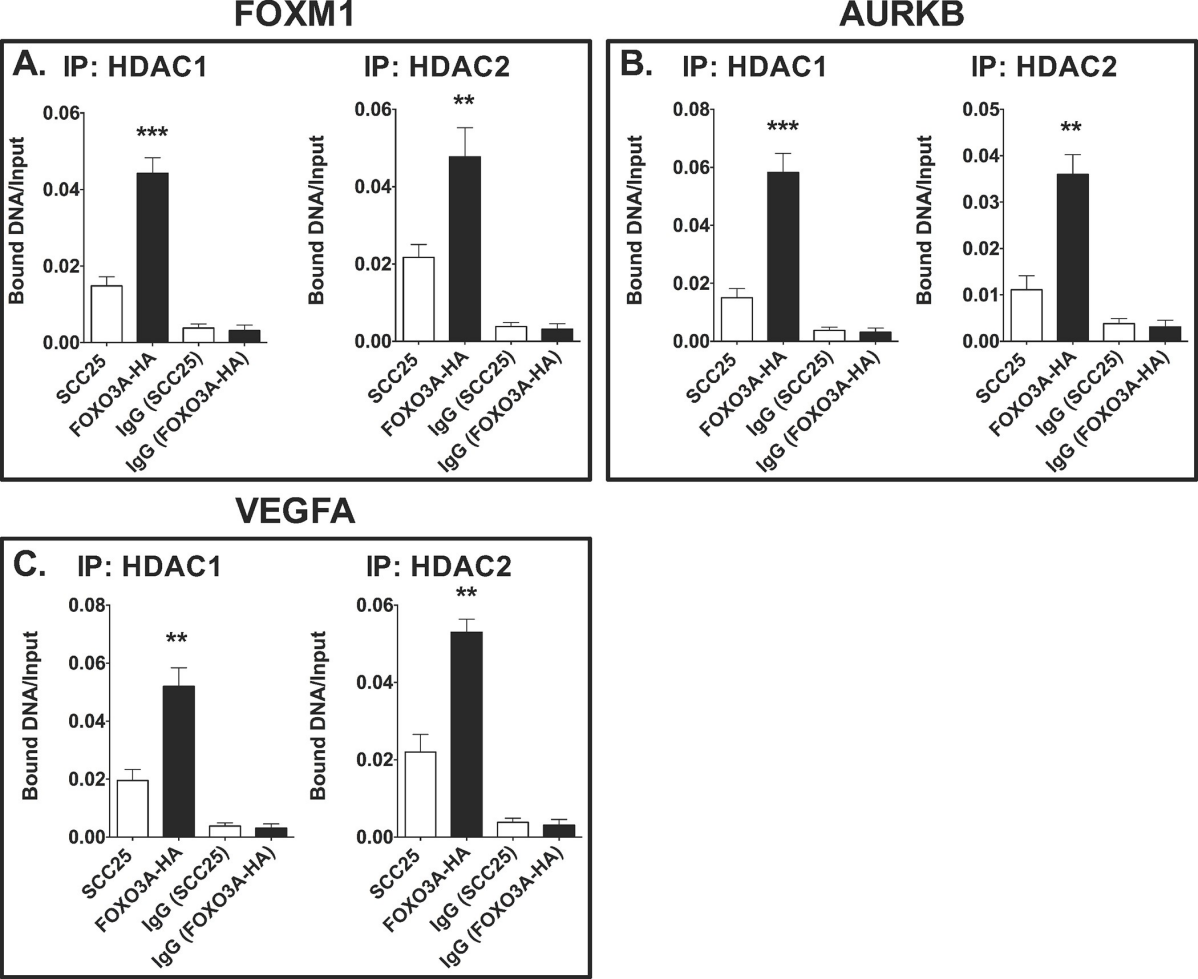
FOXO3A overexpression induces the recruitment of HDAC1 and HDAC2 to the promoters of FOXM1, Aurora kinase B, and VEGFA. The recruitment of HDAC1 (left panels) and HDAC2 (right panels) was determined by ChIP analysis in parental SCC-25 cells and FOXO3A overexpressing SCC-25 cells of the promoters of FOXM1 (A), Aurora kinase B (B), and VEGFA (C). These data are from four independent experiments ± SEM, as described in Fig 5. Statistical significance was determined by the Student’s t-test, which compared HDAC1 and HDAC2 recruitment patterns in the parental SCC-25 cell line to FOXO3A overexpressing SCC-25 cells (**, *p*<0.01, and ***, *p*<0.001).

The activity of HDACs has been implicated in the tumor suppressive properties of FOXO3A with respect to modulating target gene expression [44, 45]. Also, FOXO3A and other FOX transcription factors, such as FOXA1, FOXA2, FOXA3, FOXO1, and FOXE, are considered “pioneer” factors because they coordinate the recruitment of other transcription factors, co-activators, co-repressors, and histone modifying enzymes such as HDACs [7, 27, 46]. We next assessed the binding patterns of HDAC1 and HDAC2 on the promoters of FOXM1 (Fig 6A), Aurora kinase B (Fig 6B), and VEGFA (Fig 6C) in the SCC-25 cell lines that stably overexpress FOXO3A. We found increased recruitment of HDAC1 (*p*<0.001; Fig 6A, left panel) and HDAC2 (*p*<0.01; Fig 6A, right panel) at the FOXM1 promoter in SCC-25 cells that overexpress FOXO3A (FOXO3A-HA), compared to parental SCC-25 cells. The DNA Bound/Input ratios of HDAC1 in the parental SCC-25 cells versus SCC-25 cells that stably overexpress FOXO3A-HA were ~0.018 and ~0.06 (Aurora kinase B, *p*<0.001); and ~0.02 and ~0.05 (VEGFA, *p*<0.01) (Fig 6B and 6C; left panels). The DNA Bound/Input ratios of HDAC2 in the parental SCC-25 cells versus SCC-25 cells that stably overexpress FOXO3A were ~0.01 and ~0.035 (Aurora kinase B, *p*<0.01); and ~0.02 and ~0.05 (VEGFA, *p*<0.01; Fig 6B and 6C, right panels). Taken together, these ChIP results (Fig 6) show that FOXO3A reduces the transcription of target promoters in part by recruiting HDAC1 and HDAC2 to promoter regions of FOXM1, AURKB, and VEGFA.

## Discussion

### Retinoic acid and/or bexarotene can regulate expression of FOX transcription factors and their downstream target genes

We investigated the effects of RA and bexarotene on the transcript levels of members of the FOX transcription factor family (FOXO1, FOXO3A, and FOXM1) and selected targets of these FOX family transcription factors (AURKB, VEGFA, and TRAIL receptors 1 and 2) [7, 8]. RA, bexarotene, and the combination increased the transcript levels of FOXO1 and FOXO3A in SCC-4 and SCC-25 cells (Fig 1). A previous study demonstrated that RA reduces the phosphorylation of FOXO3A and induces its nuclear translocation to initiate TRAIL-mediated apoptosis in acute promyelocytic leukemia (APL) [47]. By microarray analysis Seo *et al*. found that bexarotene increased the transcript (and protein) levels of FOXO3A in human breast cancer cells [48]. Also, RA has been shown to increase FOXO1 expression, in a time-dependent manner, in human breast cancer [49]. Because all FOXO isoforms have an LxxLL motif, which is involved in interactions with nuclear hormone receptors, FOXO transcription factor-binding partners include several nuclear hormone receptors, such as retinoic acid receptor (RAR) and retinoid X receptor (RXR) [50]. RA, bexarotene, and the combination also reduced the transcript levels of FOXM1 (Fig 1). Consistent with the data obtained in this study, other groups have determined that RA can reduce the expression of FOXM1 in neuroblastoma [51] and in ovarian cancer [52] cells. We conclude that the FOXO1, FOXO3A, and FOXM1 transcription factors are likely targets of retinoids (retinoic acid) and rexinoids (bexarotene) in OSCC.

Since we found significant changes in the FOXO1, FOXO3A, and FOXM1 transcript levels induced by RA and/or bexarotene (Fig 1), we next wanted to determine if the expression of downstream target genes is altered by these drugs. Aurora kinase B, a gene involved in the regulation of cell proliferation [7] whose transcript levels are induced by FOXM1 [53], exhibited reduced transcript levels when OSCC-derived cells were treated with RA and RA+Bex (Fig 2A and 2E). Since VEGFA is a well-established oncogene that induces angiogenesis and is a direct target of both FOXO3A [54] and FOXM1 [55], the reduction of VEGFA transcript levels is likely to be the result of antagonism of FOXO3A and FOXM1 on the VEGFA promoter. Since RA reduces VEGFA transcript levels (Fig 2B and 2F), these data suggest that RA can reduce activators involved in oncogenesis partially by increasing and reducing the expression of FOXO3A and FOXM1, respectively.

The tumor necrosis factor-related apoptosis-inducing ligand (TRAIL) receptors have been implicated in inducing apoptosis in cancer cells [56]. Clinical studies in which these receptors were activated by monoclonal antibodies or small molecule compounds have demonstrated the importance of TRAIL receptors in various malignancies such as OSCC, non-small cell lung cancer, and metastatic colorectal cancer [57]. FOXO3A has been implicated in the regulation of other pro-apoptotic genes, such as Bim, PUMA, and FasL in multiple cancer-derived and neoplastically transformed cell lines [58]. Similar to FOXO3A’s role in pro-apoptotic mechanisms, recent studies have established that retinoic acid [59, 60], and bexarotene [61] can induce the expression of TRAIL receptors, and we detected increases in the transcript levels of these receptors with the overexpression of ectopic FOXO3A and with the co-administration of RA and bexarotene (Fig 2).

Since retinoic acid response elements (RAREs, *e*.*g*. [GGTTCA(N_5_)AGTTCA]) have not been identified within the promoter regions of FOX family members, it can be inferred that retinoids regulate FOXM1 (and other FOX protein) expression through indirect responses [32]. Thus, indirect regulation of FOXM1 by retinoids would occur because some FOXM1 transcriptional activators are directly suppressed by RA and other retinoids. Transcription factors that both bind to FOXM1 promoter regions and activate FOXM1 include but are not limited to GLI1, SOX2, STAT3, CTCF, HIF1 alpha, and E2F [62]. Retinoid-induced repression of the Hedgehog-Gli signaling pathway is an example of how a secondary retinoid response can affect the expression of FOXM1. Increased expression of GLI1 has been associated with poor prognosis of carcinomas stemming from the oral cavity [63, 64], the esophagus [65], the lung [66], and the skin [67]. While information is limited about retinoid regulation of GLI1 in OSCC, RA has been shown to reduce GLI1 activity in murine keratinocytes [68] and basal cell carcinoma [69].

Additionally, retinoids have long been implicated in regulating cell cycle events, specifically by inducing the degradation of various cyclins and cyclin dependent kinases (CDKs) (reviewed in [33]). Previous studies have reported overexpression of CDK4/6 in multiple tumor types including OSCC [70–72]. An *in vitro* systematic substrate screen in several cancer-derived cells lines concluded that CDK4/6 can phosphorylate and stabilize FOXM1 to promote entry into the S phase of the cell cycle [73]. These two findings suggest that RA has the potential to destabilize FOXM1 activity in OSCC by degrading CDK4/6. In the current study, we demonstrated that RA can modulate transcript levels and downstream activities of FOXM1; however, further investigation in OSCC-derived cell lines are necessary to determine potential retinoid-specific effects on FOXM1 expression and activity.

### Retinoic acid and bexarotene induce FOXO3A binding on FOXM1 target promoters

To gain a greater understanding of the transcriptional roles of FOXO3A and FOXM1 in OSCC, we generated SCC-25 cells that either stably overexpress exogenous FOXO3A and FOXM1 or that express almost no FOXM1 (S1A and S1B Fig). OSCC-derived cells expressing exogenous FOXO3A and shRNA constructs against FOXM1 had similar target gene expression patterns to parental cells treated with RA and/or Bex (Figs 2 and 4; S1 Fig). Additionally, opposing transcriptional patterns were observed in SCC-25 cells that overexpressed FOXM1, compared to cells stably expressing FOXO3A and shRNA-FOXM1 constructs (Figs 2 and 4; S1 Fig). We also examined the binding of FOXO3A and FOXM1 at promoters of target genes (Fig 5). Changes in target gene expression by either FOXO3A or FOXM1 can be accomplished through the binding of these transcription factors to target promoters. Since FOXO3A and FOXM1 bind to the same DNA motifs (5’-GTAAA(C/T)A-3’) found in forkhead response elements (FHREs), the binding of a specific FOX transcription factor (FOXO3A or FOXM1) could produce opposing transcriptional outputs based on the recruitment of activators or repressors associated with FOXO3A or FOXM1 [27, 74]. Previous studies using ChIP and other promoter analyses have confirmed direct binding of FOXM1 and/or FOXO proteins to the promoter regions of FOXM1, Aurora kinase B, CDC25, VEGFA, BIRC5, TRAIL receptor 1 and 2 [7]–target promoters that were included in this study. Sole somatic deletion of FOXO3 in murine endothelial cell homeostasis [75] and hematopoiesis [76] models did not yield severe phenotypic effects, indicating that functional redundancy exists among FOXO transcription factors (FOXO1, FOXO3A, and FOXO4). When CRISPR/Cas9 technology was used to knock out FOXO3A in UM-SCC-1 cells (another OSCC-derived cell line), FOXM1 transcript levels were significantly increased [28] confirming an inhibitory role of FOXO3A for FOXM1. Although we did not investigate the effects of RA and/or bexarotene in FOXO3A-deficient SCC25 cells, we speculate that FOXM1 and its target genes would still be suppressed in the absence of FOXO3A. Taken together, in the absence of FOXO3A expression, RA- and/or Bex-induced expression of FOXO1 (Fig 1) could reduce and antagonize FOXM1 promoter binding to downstream targets.

Competition for the same DNA motif-binding sites between members of the same gene family has been observed for other transcription factors, such as TCF7 and TCF7l1 (formerly known as TCF1 and TCF3, respectively) [77, 78]. Another study concluded that FOXO and FOXM1 promoter binding has antagonistic effects on the cell cycle inhibitors p21 and p27; FOXM1 promotes the degradation and FOXOs promote the activation of these inhibitors [79]. Additionally, the antagonistic relationship between FOXO and FOXM1 transcription factors has been shown by their competitive binding to insulin-like growth factor 1 (IGF1) regulatory sequences in developing cardiomyocytes [80]. Here we report a similar, antagonistic effect with respect to the binding of FOXO3A and FOXM1 on the promoters of FOXM1, Aurora kinase B, CDC25, and BIRC5 (Fig 5). On the FOXM1 and Aurora kinase B promoters, we found inverse binding of FOX3A and FOXM1 in the RA and/or bexarotene treatment versus untreated groups (Fig 5). Based on the changes in transcript levels of FOXO3A and FOXM1 induced by RA and/or bexarotene (Fig 1), increased FOXO3A binding on target promoters could result from the reduction of FOXM1 expression. Also, changes in transcriptional activity caused by FOXO3A could be induced, in part, by the recruitment of other transcriptional factors and epigenetic modifiers by FOXO3A [81].

### The overexpression of FOXO3A stimulates the recruitment of histone deacetylase (HDAC) 1 and HDAC2 to target promoters

Over 18 human histone deacetylases (HDACs) have been identified and grouped into four classes based on their yeast homologs. By deacetylating lysines on core histones and other proteins, HDACs play critical roles in epigenetic regulation and in the control of cellular functions, such as cell-cycle progression, cell differentiation, cell migration and invasion, apoptosis, and angiogenesis [82]. Briefly, HDAC1 and HDAC2 are members of Class I HDACs, which also includes HDAC3 and HDAC8 [82]. Retinoic acid has been implicated in the process of deacetylation and acetylation of histones induced by histone acetyltransferases (HATs) [83–85]. Similar to RA’s ability to recruit multiple HDACs, Li *et al*. found that bexarotene can modulate cyclin D1 transcription through the recruitment of HDAC1 in mammary epithelial cells [86].

It has been proposed that there is a dual plasticity by which FOX proteins can direct transcription. In addition to being conventional activators or repressors of gene expression, many FOX subtypes can function as pioneer transcription factors. Pioneer transcription factors, through initial binding to regulatory sequences, serve as building blocks for inducing transcription by opening condensed DNA and allowing the association of other factors involved in transcription [87]. Of the FOX family, FOXA proteins (specifically, FOXA1 and FOXA2) have been established as true pioneer factors by their ability to induce histone 3 lysine 4 (H3K4) methylation and DNA demethylation in certain cellular contexts [88]. Furthermore, FOXA1 has been shown to be a pioneer factor in steroid hormone signaling in breast cancer- and prostate cancer-derived cell lines [89, 90]. NMR studies have confirmed significant structural similarities between FOXA and FOXO transcription factors within the forkhead domain, suggesting potential similar pioneering functions for FOXO proteins [91]. Although there are a few studies confirming the pioneer nature of FOXO1 [92], information concerning FOXO3A’s role as a pioneer transcription factor is very limited. Even though FOXO3A has not been found to possess direct methylating or demethylating properties, FOXO3A binding has been linked to the recruitment of HDAC1 to the VEGFA promoter in MDA-MB-231 cells, a metastatic breast cancer cell line [27], similar to our findings in SCC-25 cells. In the SCC-25 FOXO3A #3 line, compared to the parental SCC-25 cell line, we detected increased recruitment of both HDAC1 and HDAC2 to FOXM1, Aurora kinase B, and VEGFA promoters (Fig 6). These ChIP analyses suggest that FOXO3A is a mediator of RA- and/or bexarotene-induced HDAC1 and HDAC2 recruitment to the FOXM1, Aurora kinase B, and VEGFA promoters. This could occur because more FOXO3A mRNA is present in the cells after RA and/or Bex addition (Fig 1). Further analyses of both FOXO1 and FOXO3A must be conducted to determine their capabilities as authentic pioneer transcription factors in OSCC.

We found that RA and/or bexarotene increase the transcript levels of FOXO1, FOXO3A, and TRAIL Receptor 1, and 2, and conversely reduce the transcript levels of FOXM1, Aurora kinase B, and VEGFA. Additionally, RA and/or bexarotene reduce proliferation of SCC-25 cells and alter the binding of FOXO3A and FOXM1 to the FOXM1, Aurora kinase B, CDC25, and BIRC5 promoters. Overexpression of FOXO3A reduces cell proliferation; reduces the transcript levels of oncogenic and pro-apoptotic genes; and induces the recruitment of HDAC1 and HDAC2 to the promoters of FOXM1, Aurora kinase B, and VEGFA. Also, shRNA-induced silencing of FOXM1 mimics cell proliferative properties and target gene expression found in cells that overexpress FOXO3A. Collectively, the results from this study demonstrate: the opposing effects that the FOXO3A and FOXM1 transcription factors have on each other; the regulation of these two transcription factors by RA and bexarotene; and the potential role of FOXO3A as a pioneer transcription factor in OSCC.

## Materials and methods

### Cell culture, chemicals, and antibodies

Human oral squamous cell carcinoma-derived cell lines SCC-4 (Cat. #CRL-1624, ATCC, Manassas, VA) and SCC-25 (Cat. #CRL-1628, ATCC) were cultured as described [93]. HPLC grade all-*trans* retinoic acid (RA; Cat. #R2625, Sigma, St. Louis, MO) and/or bexarotene (Bex; Cat. #SML0282, Sigma), dissolved in 100% dimethyl sulfoxide (DMSO_4_; Cat. #472301, Sigma), were added to media to a final concentration of 1 μM and 10 μM, respectively (final concentration of DMSO_4_, 0.1%).

For Western blotting (WB) and/or Chromatin immunoprecipitation (ChIP) the following antibodies were used: mouse monoclonal anti-Actin (C4) (WB; 1:40,000; Cat. #MAB1501, Lot #2145928, EMD Millipore, Billerica, MA), rabbit polyclonal anti-FOXM1 (K-19) (WB and ChIP; 1:500; Cat. #sc-500, Lot #12115, Santa Cruz Biotechnology, Inc., Dallas, TX), rabbit monoclonal anti-FOXO3A (75D8) (WB; 1:1000; Cat. #2497, Lot #6, Cell Signaling Technology, Inc., Danvers, MA), rabbit polyclonal anti-HA (ChIP; Cat. #ab9100, Lot #GR203089-1, Abcam, Cambridge, MA), rabbit polyclonal anti-HDAC1 (ChIP; Cat. #06–720, Lot #DAM1821113, EMD Millipore), and rabbit polyclonal anti-HDAC2 (ChIP; Cat. #ab7029, Lot #904782, Abcam). The following contains the validation references for the described antibodies: anti-Actin [60, 94], anti-FOXM1 [95], anti-FOXO3A [96], anti-HA [77], anti-HDAC1 [84], and anti-HDAC2 [84].

### Cell proliferation assays

SCC-4 and SCC-25 human cell lines were plated in 6-well plates at a density of 5x10^3^ cells/well in media supplemented with nothing, DMSO_4_, RA (all-*trans* retinoic acid), Bex (bexarotene), and RA+Bex. Cells were counted over a period of nine days using a Coulter Z1 electronic particle counter (Beckman Coulter, Inc., Fullerton, CA). For these assays, Day 0 represented the plating day and Day 1 represented the first count and the administration of the drugs described above. The medium was changed every two days and the cells were counted at days 1, 4, 7, and 10.

### Generation of FOXO3A overexpressing, FOXM1 overexpressing, and FOXM1 knockdown SCC-25 cells

The stable SCC-25-FOXO3A cell line was generated by co-transfecting HA-tagged human FOXO3A, which was previously cloned into pSG5 vector with expression driven by the SV40 promoter (FOXO3A-HA; Addgene Cat. #1787, Cambridge, MA) and the pLKO.1 vector (to induce puromycin resistance) at a 9:1 ratio (construct to empty vector). Human FOXM1 cDNA (Cat. #MHS6278-202756621, Clone id# 3881055, GE Healthcare Dharmacon, Lafayette, CO) was cloned into the pSG5 vector and overexpressed in SCC-25 cells as described above. The FOXM1 overexpressing shRNA constructs (Cat. #SHCLNG-NM_008021, Sigma) that target FOXM1 were cloned into the pLKO.1 vector and transduced into SCC-25 by lentiviral infection as described [77].

### Chromatin immunoprecipitation (ChIP) assays

SCC-25 cells (2x10^6^) were plated in 15 cm^2^ plates and on the following day the cells were treated for 72 hours. The culture medium was changed at 48 hours and the vehicle control and drugs were added for another 24 hours. Briefly, 20 μg of extracted DNA was subjected to the one-step ChIP protocol in which DNA/protein complexes were incubated with either 2 μg of antibody or an IgG (negative control) as in [97]. The primer pairs used for the ChIP assays are shown in S1 Table.

### RNA isolation, reverse transcription, and qRT-PCR

SCC cells (5x10^4^) were plated in 60 mm plates; 24 hours later the cells were treated as described above for 72 hours. Then, the cells were harvested in TRIzol (Cat. #15596–026, Life Technologies, Norwalk, CT) and total RNA was extracted, as described by the manufacturer. From total extracted RNA (1 μg), the qScript cDNA Synthesis Kit (Cat. #95048, Quanta Biosciences, Inc., Gaithersburg, MD) was used to generate cDNA. For quantitation, target gene fold changes were normalized to HPRT1 and to untreated cells. Primer pairs that span one intronic region can be found in S2 Table.

### Western blot analysis

We performed Western blotting of protein lysates (20 μg) extracted from SCC cell lines as described in [98]. The dilutions of the anti-HA, the anti-FOXM1, and the anti-beta Actin antibodies were 1:1000, 1:250, and 1:10000, respectively. We performed densitometry to normalize target protein levels to the levels of beta actin.

### Statistical analyses

All experiments included at least four independent biological assays (n ≥ 4) and the results are presented as mean ± SEM; the statistical analyses were performed using Prism 6.0 (GraphPad Software, Inc., La Jolla, CA). For gene expression and ChIP assays statistical differences between the various treated groups by using analysis of variance (ANOVA); and multiple comparisons between groups were determined by the Bonferroni *post-*hoc test. To assess the statistical significance of the recruitment of HDAC1 and HDAC2 in parental SCC-25 and SCC-25-FOXO3A-HA lines, the Student’s t-test was performed.

## Supporting information

S1 Fig The overexpression of FOXO3A and the silencing of FOXM1 induce changes cellular proliferation and changes in gene expression of downstream FOX targets. Representative Western blots confirm the protein levels of FOX transcription factors in SCC-25 cells expressing HA-tagged FOXO3A constructs (A) or shRNA constructs targeting FOXM1 (B). The changes in the gene expression of Aurora kinase B (C) and TRAIL receptor 2 (D) in the SCC-25 line containing constructs that drive the overexpression of FOXO3A, the overexpression of FOXM1, and the silencing of FOXM1 were determined by QRT-PCR analysis. The QRT-PCR data show the results of three independent experiments ± SEM. Post-hoc analyses show *, *p*<0.05 and **, *p*<0.01.
(TIFF)

S1 Table Primers used for chromatin immunoprecipitation (ChIP) assays. (XLSX)

S2 Table Primers used for QRT-PCR. (XLSX)
