# Supplementary figures and images for: Retinoids induce antagonism between FOXO3A and FOXM1 transcription factors in human oral squamous cell carcinoma (OSCC) cells
Source: PLoS One. 2019 Apr 12;14(4):e0215234. doi: 10.1371/journal.pone.0215234 (PMC6461257; doi:10.1371/journal.pone.0215234)

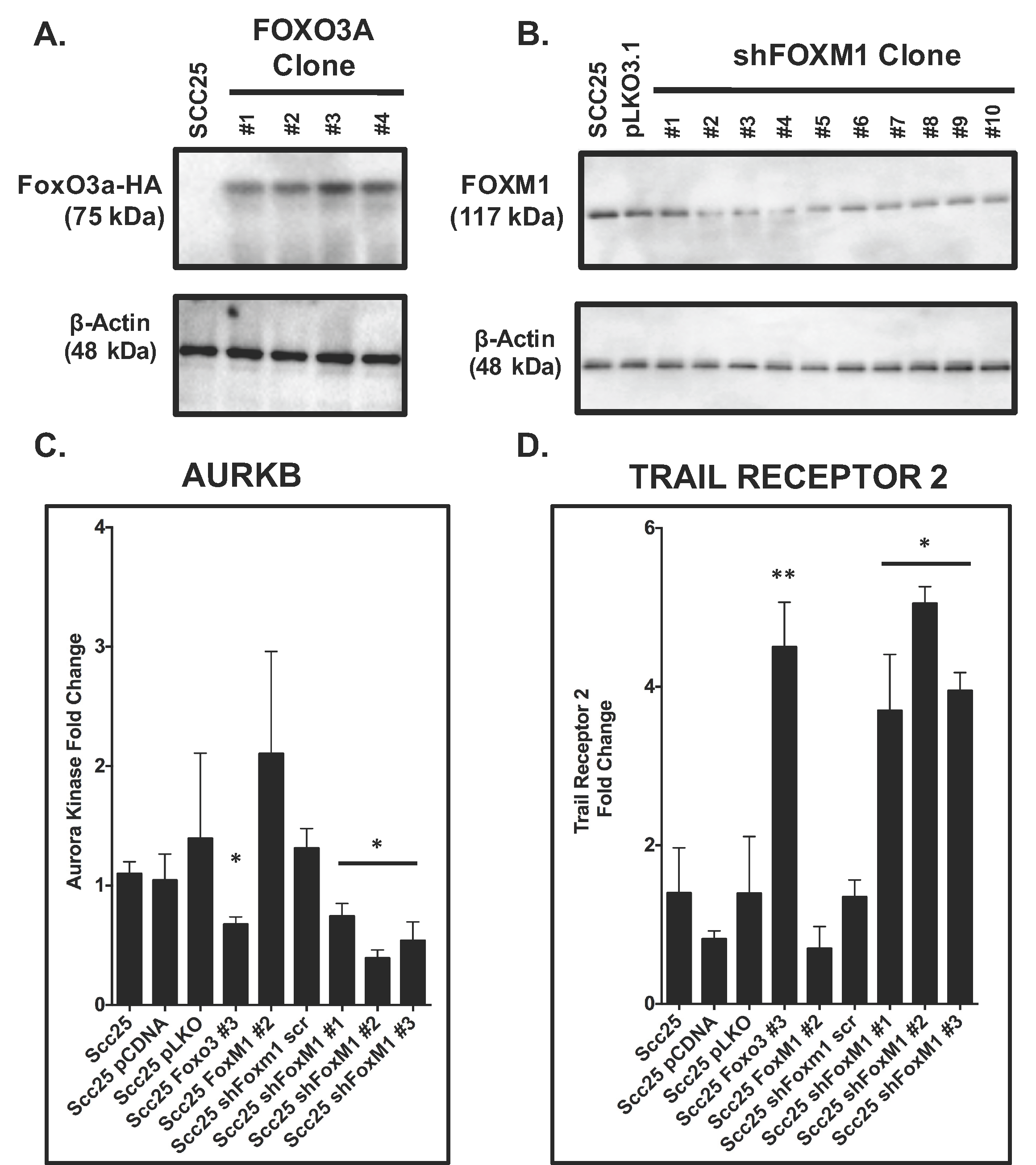

Supplement: S1 Fig — Representative Western blots confirm the protein levels of FOX transcription factors in SCC-25 cells expressing HA-tagged FOXO3A constructs (A) or shRNA constructs targeting FOXM1 (B). The changes in the gene expression of Aurora kinase B (C) and TRAIL receptor 2 (D) in the SCC-25 line containing constructs that drive the overexpression of FOXO3A, the overexpression of FOXM1, and the silencing of FOXM1 were determined by QRT-PCR analysis. The QRT-PCR data show the results of three independent experiments ± SEM. Post-hoc analyses show *, p<0.05 and **, p<0.01. (TIFF) [file pone.0215234.s001.tiff]
